# MPT64 antigen detection test improves diagnosis of pediatric extrapulmonary tuberculosis in Mbeya, Tanzania

**DOI:** 10.1038/s41598-021-97010-2

**Published:** 2021-09-02

**Authors:** Erlend Grønningen, Marywinnie Nanyaro, Lisbet Sviland, Esther Ngadaya, William Muller, Lisete Torres, Sayoki Mfinanga, Tehmina Mustafa

**Affiliations:** 1 Department of Global Public Health and Primary Care, Centre for International Health, University of Bergen, 5020 Bergen, Norway; 2 Department of Thoracic Medicine, Haukeland University Hospital, 5021 Bergen, Norway; 3 Muhimbili Medical Research Centre, National Institute for Medical Research, Dar es Salaam, United Republic of Tanzania; 4 Department of Clinical Medicine, Faculty of Medicine, University of Bergen, 5020 Bergen, Norway; 5 Department of Pathology, Haukeland University Hospital, Bergen, Norway; 6 Mbeya Zonal Referral Hospital, Mbeya, United Republic of Tanzania

**Keywords:** Infectious-disease diagnostics, Immunohistochemistry, Paediatric research, Tuberculosis, Risk factors, Pathology

## Abstract

Pediatric extrapulmonary tuberculosis (EPTB) is a diagnostic challenge. A new immunochemistry based MPT64 antigen detection test has shown improved sensitivity compared to current laboratory tests. The aim of this study was to implement and validate the test performance in a resource limited African setting. Presumptive pediatric (0–18 y) EPTB patients were prospectively enrolled at Mbeya Zonal Referral Hospital, and followed to the end of treatment or until a final diagnosis was reached. Specimens from suspected sites of infection were subject to routine diagnostics, GeneXpert MTB/RIF assay and the MPT64 test. The performance of the tests was assessed using mycobacterial culture as well as a composite reference standard. 30 patients were categorized as TB cases, 31 as non-TB cases and 2 were uncategorized. In the TB group, the three most common infections were adenitis (30%), peritonitis (30%) and meningitis (20%). The sensitivity, specificity, positive predictive value, negative predictive value and accuracy of the MPT64 test was 92%, 88%, 87%, 92% and 90%, respectively. Mortality was equally high among TB/non-TB cases (23% vs 21%), and malnutrition was the main comorbidity among TB cases. The MPT64 test was implementable in the routine diagnostics in a low-resource setting and improved the diagnosis of pediatric EPTB.

## Introduction

Diagnosing extrapulmonary tuberculosis (EPTB) in children is a challenge. The paucibacillary nature of the disease, the nonspecific clinical presentation, the need for invasive sampling and poor performance of current diagnostic tools such as mycobacterial (Mtb) culture, acid fast bacilli staining (AFB) and GeneXpert MTB/RIF assay (Cepheid, Sunnyvale, California, United States -GeneXpert) adds to the challenge. The current gold standard test, Mtb culture, does not have a perfect sensitivity, has a long turnaround time and has technical and logistical challenges, but is necessary for drug susceptibility testing. The World Health Organization (WHO) has endorsed the use of GeneXpert for EPTB samples^1^. A recent Cochrane review found varying sensitivity of GeneXpert across different EPTB samples with high specificity^2^. AFB is of limited diagnostic value^3^. Most nucleic acid amplification tests, other than GeneXpert, have better sensitivity than Mtb culture, but are complex, technically demanding, and have the risk of contamination^3^. Furthermore, they are unable to separate between dead and viable bacteria and, except from GeneXpert, gives no drug susceptibility results, all limiting their use in the resource constrained setting^3^. Fine needle aspiration cytology (FNAC) of mass lesions has high sensitivity, but it is often difficult to distinguish tuberculous lesions form other granulomatous conditions, non-tuberculous mycobacteria and atypical lesions in advanced human immunodeficiency virus (HIV) disease, thereby reducing its specificity^3^. Histology sampling is invasive and is done less in children and lacks specificity. As a consequence few pediatric EPTB cases are microbiologically confirmed^4^, and empirical treatment with anti-tuberculosis therapy for presumptive EPTB is often started. This can lead to both over- and undertreatment, thus contributing to excess mortality and morbidity. There is a clear need for better diagnostic tools that are robust, precise and implementable in a low resource setting.

Immunostaining using anti-MPT64 has shown to have comparable sensitivity and specificity to nested polymerase chain reaction^3,5^. MPT64 is a protein secreted by the mycobacterium tuberculosis complex species. It is not detected in non-tuberculous mycobacteria and Bacillus Calmette-Guerin strains with RD2 deletion^6^. A recent study in Zanzibar found a sensitivity of 100% for TB adenitis in children when comparing the anti-MTP64 test (MPT64 test) to a composite reference standard^7^.

Tanzania is one of 30 high burden TB countries^8^. In 2018 EPTB constituted 20% of the 75,828 notified TB cases, and 14% of all new TB cases were pediatric. 28% of all new TB cases were HIV infected in 2018^9^. Mbeya region in the Southern Highlands of Tanzania, has an HIV prevalence of 9.3% among adults (> 15 years old), with an estimated pediatric (< 15 y) prevalence of 0.5%^10^. The National Nutrition Survey estimates a prevalence of severe and moderate acute malnutrition of 2.7% in children < 5 years in Mbeya region^11^. Mbeya Zonal Referral Hospital is a tertiary hospital, and serves approximately 8 million people in six regions in the Southern Highlands.

The aim of this study was to assess if the MPT64 test was implementable in the routine TB care for children at Mbeya Zonal Referral Hospital, and to compare the performance of the test to the routine diagnostic tests including GeneXpert.

## Materials and methods

### Recruitment of patients, data collection and follow up

**Figure 1:**
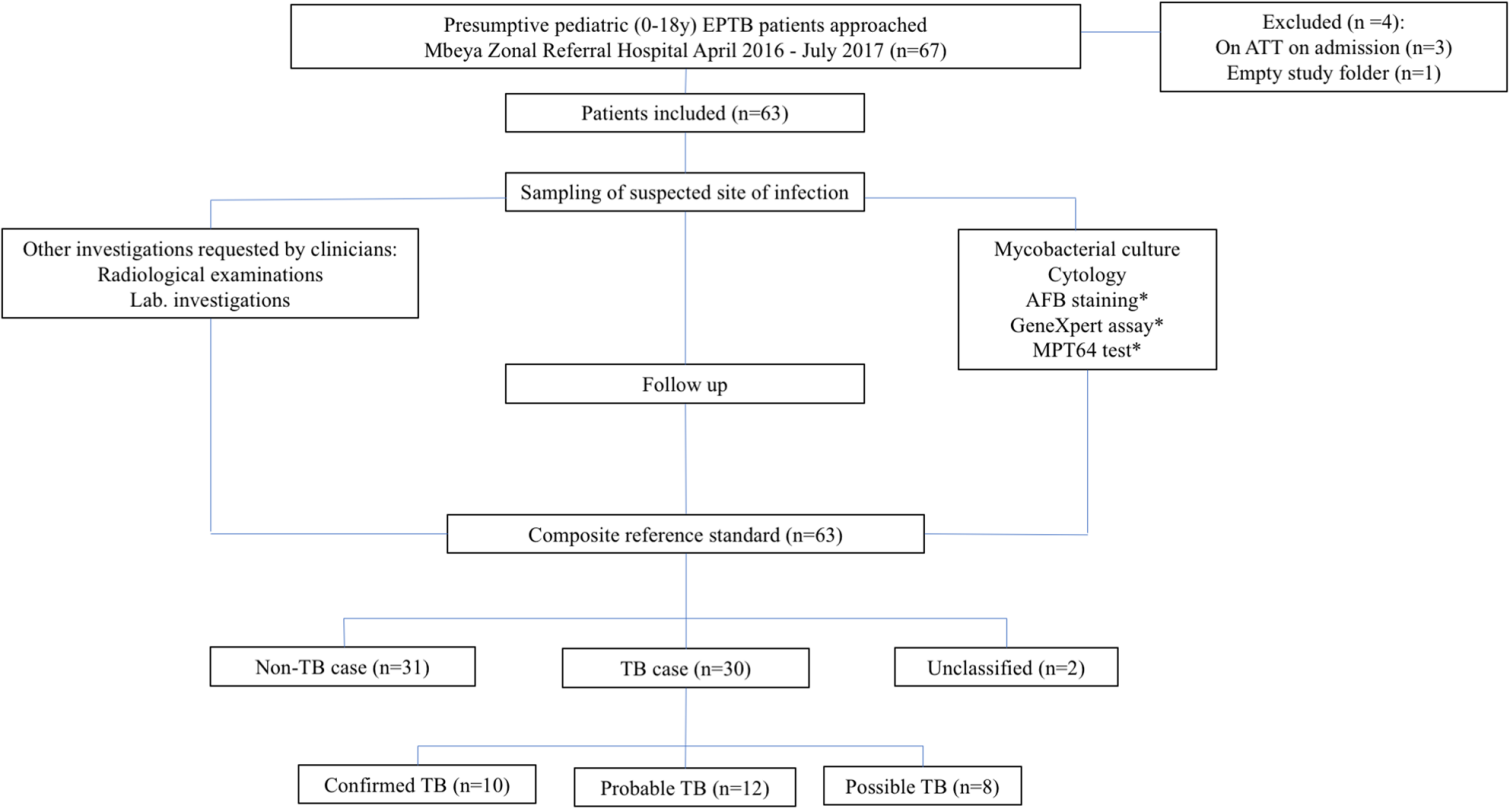
Flowchart of inclusion of patients and study design. *Not part of composite reference standard.

The study was part of a larger study conducted at Mbeya Zonal Referral Hospital to validate the MPT64 antigen detection test. The study design is shown in Fig. 1. Children (0–18 y) with suspected EPTB from both the in- and outpatient services were prospectively enrolled between April 1st 2016 and July 31st 2017. Clinicians were asked to recruit patients with suspected EPTB from both in-and outpatient services. The study protocol did not give any guidance to the clinicians with regards to which symptoms/findings that were compatible with EPTB. Mbeya Zonal Referral Hospital mainly receives referrals from other clinics.

Relevant clinical data was collected through a questionnaire previously developed and validated in a similar study in Zanzibar^7^. To assess the validity of the questions, the translation of the questions between English/Swahili was done by two separate bilinguals. Any ambiguity in the questions was assessed and corrected after a test among three patients in the Zanzibar study. The questionnaire included questions about sociodemographic characteristics, health seeking behavior, TB knowledge and beliefs and previous medical history. Information about HIV status was obtained from the patient disclosure, and additional HIV testing was performed only per request of the clinicians as per standards of care. Further, radiological examinations and biochemistry was prescribed by the clinicians. Patients who did not consent or had received TB treatment within the last 12 months were excluded. Data collection and clinical examination was done by medical officers hired for the purpose of the study.

Patients were followed up at 2–3 months, at the end of treatment, or until a diagnosis other than EPTB was reached. If the patients had been marked as lost to follow up, a new tracing was performed. In addition, a review of electronic hospital records was done both at Mbeya Zonal Referral Hospital and Baylor College of Medicine Children´s Foundation Tanzania (Baylor) for completeness of clinical data. Baylor provides comprehensive care and treatment for pediatric tuberculosis, HIV/AIDS, malnutrition and other complicated or chronic pediatric conditions in the region.

### Diagnostic samples and laboratory tests

Samples were taken from suspected site of infection by the pathologists. Materials from superficial, palpable masses and enlarged lymph nodes were collected using a 22 gauge needle attached to a syringe, while applying minimal negative pressure. The material obtained was smeared on to glass slides for cytological examination, MPT64 staining and Ziehl–Neelsen staining (ZN). Sterile saline was used to rinse the needle and syringe to obtain specimens for Mtb culture and GeneXpert.

### Diagnostic tests

Microscopy for AFB was done using ZN. Mtb cultures and GeneXpert samples were sent to the Central Tuberculosis Reference Laboratory located at the Muhimbili National Hospital in Dar es Salaam. Samples were sent in a cool box within two days after sampling using public transportation bi-weekly. The samples were stored at 4 degrees before transportation. Culture was performed on Lowenstein-Jensen medium according to the standard protocol of the Central Tuberculosis Reference Laboratory. GeneXpert was also performed according to the protocol of the Central Tuberculosis Reference Laboratory, in accordance with the WHO protocol.

Cytological slides were stained with the Papanicolaou stain, histological slides with haematoxylin and eosin. On FNAC granulomatous inflammation with/without necrosis or any necrosis (excluding suppurative inflammation) was defined as compatible with TB^12^. Biopsy findings compatible with TB were defined as the same as the FNAC findings. On cytology, finding of predominantly lymphocytes and/or macrophages, or epitheloid cells and/or necrosis was defined as compatible with TB^13–17^.

**Figure 2:**
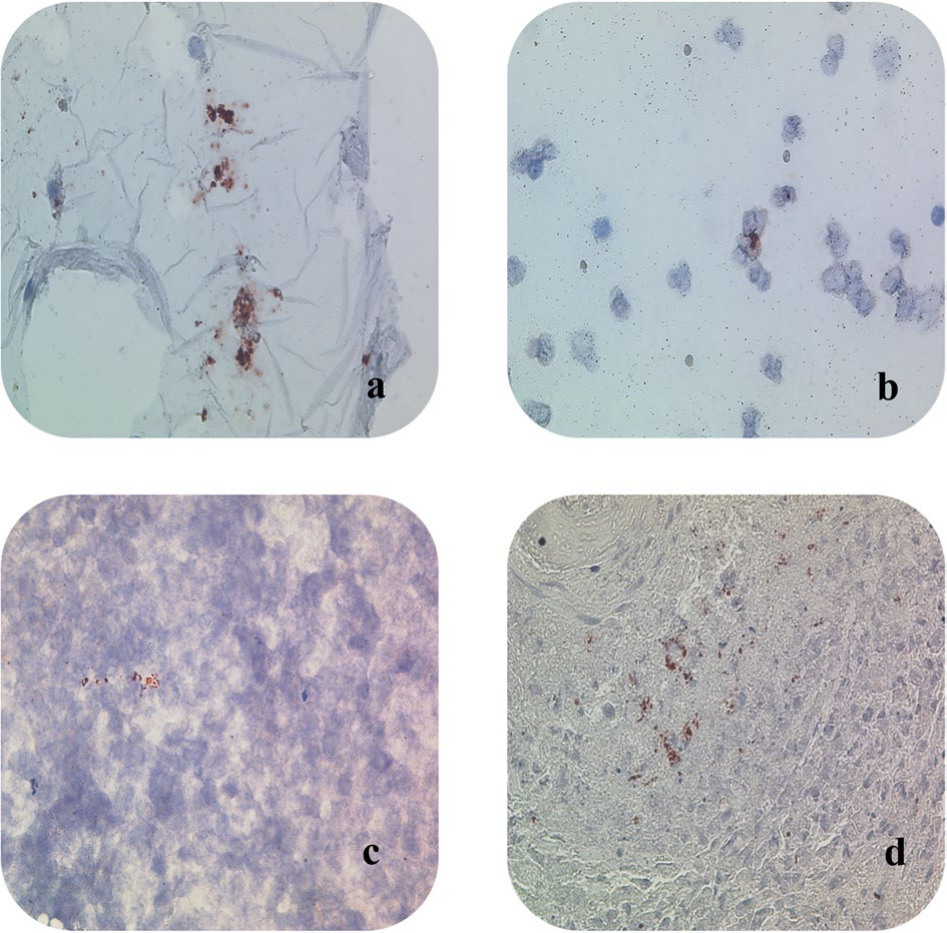
Positive MPT64 staining (red-brown color) in TB cases. Picture (**a**) MPT64 immunostained smear from pleural effusion showing positive spots (red-brown) within macrophages (X40). Picture (**b**) MPT64 immunostained smear of CSF showing positive spots within macrophages(X40). Picture (**c**): MPT64 immunostained smear from FNAC of lymph node. Positive spots for MPT64 are seen and the background shows extensive caseous necrosis (X40). Picture (**d**) MPT64 immunostained histological section of lymph node of adult patient. Positive spots for MPT64 within cytoplasm of macrophages and background showing granuloma formation and necrosis (X40).

Two technicians at MZRH were trained in the procedure of immunocytochemistry/immunohistochemistry staining (immunostaining). Two pathologists (WM and LT) received training in evaluating immunostaining. The previously alcohol fixed smears were hydrated through decreasing grades of alcohol, washed in distilled water, and incubated with hydrogen peroxide to inhibit endogenous peroxidase activity. Immunostaining was performed as described previously^18,19^, but with some modification^7^, by using an in-house polyclonal anti-MPT64 antibody at 1/250 dilution and Dako kit (Dako Envision + System-HRP, K4009, Dako, Glostrup, Denmark) to demonstrate the MPT64 antigens. The primary antibody was then applied and incubated, as described previously^7^. The immunostained slides were assessed by the local pathologist (WM and LT). The pathologists were blinded for the results of Mtb culture and GeneXpert. The immunostaining was regarded as positive if reddish granular intracytoplasmic staining or extracellular staining was found in necrotic areas, as shown in Fig. 2.

## Definitions

### Categorization of patients

Patients were classified into the confirmed TB, probable TB, possible TB and not TB groups according to a composite reference standard (CRS), as shown in Table 1.

**Table 1:** Criteria for categorizing patients-composite reference standard.

Confirmed TB case	Positive mycobacterial culture on extrapulmonary sample
Probable TB case	Clinically presumptive EPTB and a response to TB treatment at 2–3 months and/or end of treatment or clinically presumptive EPTB and bacteriologically confirmed concomitant Pulmonary TB^a^ and one of the following Radiological findings suggestive of EPTB^b^ Effusions/CSF: cytological findings consistent with TB^c^ FNAC/biopsy: morphological features consistent with TB^d^
Possible TB case	Strong clinical suspicion of EPTB and/or patient started TB treatment and one of the following Response to TB treatment at 2–3 months and/or end of treatment Radiological findings suggestive of EPTB Effusions/CSF: cytological findings consistent with TB FNAC/biopsy: morphological features consistent with TB
Non-TB case	Negative mycobacterial culture and one of the following Recovery without TB treatment and/or after specific non-tuberculous therapy Cytology/histology examination or review of clinical records concluded other diagnosis than TB TB treatment completed for presumptive EPTB without improvement
Uncategorized patient	Not possible to categorize according to composite reference standard

EPTB, extrapulmonary tuberculosis; TB, tuberculosis; CSF, cerebrospinal fluid; FNAC, fine-needle aspiration cytology; ZN, Ziehl–Neelsen.

^a^Positive sputum by Mycobacterial culture, GeneXpert or ZN.

^b^Pleural effusion and/or infiltrates/nodules consistent with tuberculosis noted on chest x-ray, signs of skeletal TB present or CT findings compatible with TB.

^c^Findig of predominantly lymphocytes and/or macrophages, or epitheloid cells and/or necrosis.

^d^Finding of granulomatous inflammation with/without necrosis or any necrosis (excluding suppurative inflammation).

### Malnutrition, immunosuppression and severity of EPTB manifestations

Severe and moderate acute malnutrition was defined by the clinicians in Mbeya Zonal Referral Hospital and Baylor using WHO 2006 growth references^11^. Severe and moderate cases were merged as ´Malnutrition´ for analysis. HIV infected patients, patients with suspected hematological malignancies, lymphomas and patients on immunosuppressive drugs were grouped together as ´Immunosuppression´ for statistical analysis. For statistical analysis TB adenitis was labelled as ´non-severe TB´ and all other EPTB manifestations were labelled ´severe-TB´.

### Ethical considerations

The project was approved by the Regional Committee for Medical Research Ethics in Norway, REK Helse-Vest (2014/46/REK vest), and the Ethical committee for biomedical research at the National Medical Research Coordinating Comittee in Tanzania with ethical clearance reference number NIMR/HQ/R.8a/Vol.IX/2142. All experiments were performed in accordance with relevant guidelines and regulations. Written informed consent and assent (for under 18 y) were obtained from all patients or caretakers. Invasive sampling was done per request of the clinicians based on patient management, not primarily for study purposes. Patients were HIV tested routinely, and not for the purpose of the study.

### Statistical analysis

Statistical analysis was performed using Statistical Package for the Social Sciences (SPSS) for Mac, version 25.0. Chi square test was used to assess differences in categorical variables. Not normally distributed variables were assessed using non-parametric tests. Sensitivity, specificity, positive predictive value (PPV), negative predictive value (NPV) and accuracy was calculated using cross-tabulation. A *p*-value < 0.05 was considered as statistically significant.

## Results

### Patient characteristics

A total of 63 patients were included in the study, as shown in Fig. 1. Per the CRS, 30 (49%) patients were classified as TB cases; 10 (33%) confirmed, 12 (40%) probable and 8 (27%) possible, respectively. 31 (51%) patients were classified as non-TB cases. 2 cases were unclassifiable and were excluded from further analysis.

Table 2 shows the baseline characteristic of the two groups. The median age in the TB and non-TB group was 8 and 10 years, respectively. There was not a significant difference in gender distribution among the two groups, but within the TB group the significant majority of cases were male (70%). A statistically significant higher proportion of patients in the TB group were recruited as inpatients.

**Table 2:** Baseline and demographic characteristics of the 61 study participants.

	TB (N = 30)	Non-TB (N = 31)	*p*-value
Age yrs median-range	8 (2 m–18 y)	10 (1y–18y)	.291 +
**Age groups**			.869
0–5	11 (37%)	12 (39%)	
6–18	19 (63%)	19 (61%)	
**Sex**			.222
Female	9 (30%)	14 (45%)	
Male	21 (70%)	17 (55%)	
**In-/outpatient**			.020
Inpatient	24 (80%)	16 (52%)	
Outpatient	6 (20%)	15 (48%)	
**BCG vaccination**			1.000
Yes	27 (90%)	27 (87%)	
No	1 (3%)	1 (3%)	
Unknown^a^	2 (7%)	3 (10%)	
**Susp. site of disease**			
Adenitis	9 (30%)	18 (58%)	
Pleuritis	2 (7%)	2 (7%)	
Peritonitis	9 (30%)	1 (3%)	
Meningitis	6 (20%)	5 (16%)	
Osteomyelitis	0	1 (3%)	
Mastitis	0	1 (3%)	
Multiorgan involvement^b^	2 (7%)	3 (10%)	
Pulmonary with EPTB	2 (7%)	0	
**Severity of disease**^**c**^			0.027
Severe disease	21 (70%)	13 (42%)	
Non-severe	9 (30%)	18 (58%)	
**HIV status**			.625
Positive	5 (17%)	3 (10%)	
Negative	25 (83%)	22 (71%)	
Unknown^a^	0	6 (9%)	
**Malnutrition**			.016
Yes^d^	13 (43%)	4 (13%)	
No	17 (57%)	25 (81%)	
Unknown^a^	0	2 (7%)	
**Any immunosuppresion**			.263
Yes^e^	19 (63%)	14 (45%)	
No	11 (37%)	16 (52%)	
Unknown^a^	0	1 (3%)	
**ATT given**			.000 (y/n *p* value)
Yes	26 (87%)	3 (10%)	
Mortality during treatment	4/26	–	
Response to treatment	18/26	–	
No response to treatment	2/26	–	
Lost to follow up	2/26	–	
No	4 (13%)	27 (87%)	
Unknown	0	1 (3%)	
**Deaths from any cause before or during TB treatment**^f^	6/26 (23%)	4/19 (21%)	.872
Lost to follow up	4/30 (13%)	12/31 (39%)	.024

^a^Unkown removed from *p*-value analysis.

^b^Includes 3 cases with suspected EPTB in 2 sites, 1 suspected military TB and 1 disseminated TB.

^c^Non severe TB: TB Adenitis. Severe TB: All other EPTB forms.

^d^In TB group: 12 severe acute malnutrition (SAM), 1 moderate acute malnutrition (MAM). Non-TB group: 2 SAM, 2 MAM.

^e^Includes SAM/MAM, HIV infected, susp. hematological malignancies (N = 2), lymphomas (N = 8) and immunosuppressive drugs (corticosteroids) (N = 1). There is overlap in the groups.

^f^Patients lost to follow up removed from analysis.

+ Mann–Whitney test. Other *p*-values chi square test.

The sites of suspected disease were heterogeneous. In the TB group, the three most common infections were adenitis (30%), peritonitis (30%) and meningitis (20%). Non-severe disease was more prevalent than severe disease in the non-TB group.

Overall, 8/55 (15%) patients were HIV positive. There was a non-significant difference in numbers of HIV infected between the TB and non-TB group. Malnutrition was the main comorbidity, with 17 patients (29%) being malnourished. Malnutrition was more prevalent among TB patients, with a prevalence of 43% vs 13% in the TB and the non-TB patients, respectively.

The two major non-TB diagnosis were malignancies (11/31) and other infectious conditions (10/31). Among other infectious conditions abscesses (4/31), bacterial meningitis (2/31), and one case each of pneumonia with empyema (1/31), cryptococcal meningitis (1/31), chronic otitis media (1/31) and suppurative lymphadenitis (1/31), were found. Benign tumors (3/31), unspecific lymphadenopathy (3/31) and undefined (4/31) were also found.

### Impact of malnutrition and immunosuppression on disease severity

The patients with malnutrition had a statistically significant higher prevalence of TB than those without malnutrition (76% vs 40%, respectively, *p* = 0.012). The malnourished patients were significantly younger (median 4 y vs 11 y, *p* = 0.003)). The patients with malnutrition had a trend towards more severe sites of infection (76% vs 50%) and higher mortality (36% vs 16%), however both trends were not statistically significant.

Among the malnourished patients, the final diagnosis was EPTB (13/17); peritonitis (4/17), meningitis (4/17), adenitis (2/17), disseminated TB (2/17), pulmonary TB with concomitant adenitis (1/17), and non-TB (4/17); hematological malignancies (2/17), lymphoma (1/17) and bacterial meningitis (1/17).

Immunosuppression in some form was present in 33 patients (55%). The group of immunosuppressed did not differ significantly with age, gender, disease site severity or mortality when compared to patients without immunosuppression. However, there was a trend towards increased mortality (32% vs 10%, *p* = 0.078) in the immunosuppressed group.

Among the immunosuppressed patients, the final diagnosis was EPTB (19/33), malignancies (10/33), unspecific lymphadenopathy (2/33) and infectious conditions (2/33).

### Mortality

The mortality did not differ significantly between the TB (23%) and non-TB (21%) groups. TB meningitis (3/10), TB pleuritis (2/10), TB peritonitis (1/10) caused mortality in the TB group, and lymphoma (2/10), hematological malignancy (1/10) and cryptococcal meningitis (1/10) in the non-TB group. Severe-TB and hospitalization were significantly associated with increased mortality by using chi-square. However, a logistic regression analysis of various factors associated with mortality did not find any variables to be statistically significant.

### Diagnostics tests

Table 3 shows the performance of all diagnostic tests for different samples. The MPT64 test performed better than Mtb culture, GeneXpert and ZN staining, with a sensitivity of 92%. No samples were positive with ZN staining. Specificity of the MPT64 test was lower than the other diagnostic tests, but acceptable at 88%.

**Table 3:** Positive test results across different specimens and in immunosuppression and malnutrition.

Samples	ZN^a^	Culture^b^	GeneXpert^c^	MPT64^d^
**TB samples (n = 30)**	0/26 (0%)	10/22 (45%)	9/29 (31%)	24/26 (92%)
Lymph node (n = 11)	0/8 (0)	2/6 (33)	2/11 (18)	10/10 (100)
FNA (10)	0/7 (0)	2/6 (33)	2/10 (20)	10/10 (100)
Biopsy (1)	0/1 (0)	0	0/1	0
Ascites (10)	0/9 (0)	4/8 (50)	3/10 (30)	6/8 (75)
Pleural effusion (3)	0/3 (0)	2/3 (66)	2/3 (66)	3/3 (100)
CSF (6)	0/6 (0)	2/5 (40)	2/5 (40)	5/5 (100)
**NON-TB samples (n = 31)**	0/23 (0)	0/27 (0)	1/30 (3)	2/16 (13)
Lymph node (20)				
FNAC (20)	0/14 (0)	0/16 (0)	1/19 (5)	2/11 (18)
Ascites (1)	0/1 (0)	0/1 (0)	0/1 (0)	0
Pleural effusion (4)	0/3 (0)	0/4 (0)	0/4 (0)	0/2 (0)
CSF (5)	0/4 (0)	0/5 (0)	0/5 (0)	0/3 (0)
Breast biopsy (1)	0/1 (0)	0/1 (0)	0/1 (0)	0
**Results in Immunosuppression**				
TB samples (n = 30)				
Immunosuppression (19)	0/16 (0)	5/13 (38)	5/18 (28)	14/15 (93)
Malnutrition (13)	0/11 (0)	3/10 (30)	3/12 (25)	9/10 (90)
Non-TB samples (n = 31)				
Immunosuppression (14)	0/8 (0)	0/12 (0)	1/13 (8)	1/7 (14)
Malnutrition (4)	0/2 (0)	0/4 (0)	0/4 (0)	0/1 (0)
Unknown immune status (1)	0/1 (0)	0/1 (0)	0/1 (0)	0/1 (0)

^a^ZN:10 missing samples, 2 inconclusive samples. 8 missing in non-TB group, 2 missing and 2 inconclusive in TB group.

^b^Culture: 2 missing samples, 10 contaminated. 1 missing and 7 contaminated in TB-group, 1 missing and 3 contaminated in non-TB group.

^c^GeneXpert:2 missing samples. 1 in TB group, 1 in non-TB group.

^d^MPT64: 16 missing samples, 3 uncertain test results. 4 missing and 0 uncertain in TB group, 12 missing and 3 uncertain (1 pleural effusion, 1 ascites, 1 lymph node FNAC) in non-TB group.

Table 4 shows the 95% C.I., sensitivity, specificity, positive predictive value (PPV), negative predictive value (NPV) and accuracy for all tests. The MPT64 test had a comparable PPV to GeneXpert, but a higher NPV than both Mtb culture and GeneXpert. The accuracy of the MPT64 test was higher than the other test. FNAC had a comparable sensitivity and specificity to the MPT64 test.

**Table 4:** Diagnostic validation of tests for EPTB.

	Sensitivty (95% C.I)	Specificity (95% C.I)	Positive predictive value (95% C.I)	Negative predictive value (95% C.I)	Accuracy (95% C.I)
**CRS defined EPTB (n = number of samples)**					
ZN (N, TB = 26, non-TB = 23)	0 (0–13)	100 (85–100)	–	51 (51–51)	51 (36–66)
Culture (TB = 22, non-TB = 27)	45 (24–68)	100 (87–100)	100	66 (57–74)	73 (59–85)
GeneXpert (TB = 29, non-TB = 30)	31 (15–51)	97 (83–100)	90 (55–99)	59 (53–65)	65 (51–77)
MPT64 (TB = 26, non-TB = 16)	92 (75–99)	88 (62–98)	87 (66–96)	92 (76–98)	90 (77–97)
FNAC + (TB = 10, non-TB = 20)	90 (56–100)	95 (75–100)	95 (72–99)	91 (61–98)	93 (77–99)
**Culture confirmed**^**a**^					
ZN (TB = 9, non-TB = 40)	0 (0–34)	100 (91–100)	–	84 (84–84)	84 (70–93)
GeneXpert (TB = 10, non-TB = 40)	80 (44–97)	98 (87–100)	87 (48–98)	96 (88–99)	95 (85–99)
MPT64 (TB = 10, non-TB = 28)	90 (56–100)	60 (39–79)	31 (21–43)	97 (82–100)	65 (47–80)
FNAC (TB = 2, non-TB = 29)	100 (16–100)	72 (53–87)	42 (28–56)	100	77 (58–90)
**Test results in Immunosuppression**^**b**^					
ZN (TB = 16, non-TB = 8)	0 (0–21)	100 (63–100)	–	43 (43–43)	43 (23–64)
Culture (TB = 13, non-TB = 12)	38 (14–68)	100 (74–100)	100	55 (44–65)	65 (43–83)
GeneXpert (TB = 18, non-TB = 12)	28 (10–53)	92 (64–100)	83 (39–97)	49 (41–57)	55 (36–73)
MPT64 (TB = 15, non-TB = 7)	93 (68–100)	86 (42–100)	90 (59–98)	90 (58–98)	90 (70–99)
FNAC (TB = 7, non-TB = 11)	100 (59–100)	100 (72–100)	100	100	100 (81–100)
**Test results in Malnutrition**^**c**^					
ZN (TB = 11, non-TB = 2)	0 (0–28)	100 (16–100)	–	24 (24–24)	24 (5–54)
Culture (TB = 10, non-TB = 4)	30 (7–65)	100 (40–100)	100	31 (23–40)	46 (20–74)
GeneXpert (TB = 12, non-TB = 4)	25 (5–57)	100 (40–100)	100	29 (23–36)	42 (19–69)
MPT64 (TB = 10, non-TB = 1)	90 (56–100)	100 (3–100)	100	75 (32–95)	92 (61–100)
FNAC (TB = 4, non-TB = 3)	100 (40–100)	100 (29–100)	100	100	100 (59–100)

^a^Culture negatives cases are added to non-TB cases. Non-TB group consists of the CRS defined cases and the previous non-TB group. Disease prevalence set to 10/61 (culture confirmed/whole cohort).

^b^Specificity calculated for immunosuppressed in the non-TB group. The disease prevalence is set to 19/33 (EPTB cases among immunosuppressed/ total immunosuppressed).

^c^Specificity calculated for malnourished in the non-TB group. The disease prevalence is set to 13/17 (EPTB cases among malnourished/ total malnourished).

Of the 30 TB cases, 26 had samples that were analyzed with the MPT64 test. Only 2 (8%) were negative; both of these tests were performed on ascites, giving a sensitivity of 75% (6/8) for ascites. One of these samples was positive for both Mtb culture and GeneXpert, and this is the only sample that had a discordant result of MPT64 and Mtb culture/GeneXpert. All remaining 24 TB cases examined with the MPT64 test were positive.

Table 5 show the correlation between diagnostic tests and findings on FNAC and cytology. The MPT64 test had a good correlation with positive findings on FNAC. The MPT64 test also had a better correlation with cytology findings compatible with TB than Mtb culture and GeneXpert.

**Table 5:** Positive test results and their correlation to cytomorphological features on FNA and cytological findings in effusions.

**FNAC (n = 32)**	**TB (n = 11)**					**NON-TB (n = 21)**				
	Cytology	ZN	Culture +	GeneXpert +	MPT64 +	Cytology	ZN	Culture +	GeneXpert +	MPT64 +
**Epitheloid cells + / − mixed inflammatory cells and + / − necrosis**	6/10 (60%)	0/3 (0)	2/4 (50%)	1/6 (17%)	6/6 (100%)	0	0	0	0	0
**Mixed inflammatory cells with necrosis**	3/10 (30%)	0/3 (0)	0/1 (0)	1/3 (33%)	3/3 (100%)	1/21 (5%)	0/1 (0)	0/1 (0)	0/1 (0)	1/1 (100%)
Suppurative inflammation + / − necrosis	1/10 (10%)	0/1 (0)	0/1 (0)	0/1 (0)	1/1 (100%)	3/21 (14%)	0/3 (0)	0/3 (0)	0/3 (0)	0/1 (0)
Reactive lymph node hyperplasia	0	0	0	0	0	2/21 (10%)	0/1 (0)	0/2 (0)	0/2 (0)	0/1 (0)
Lymphoma	0	0	0	0	0	6/21 (29%)	0/3 (0)	0/4 (0)	0/5 (0)	1/4 (25%)
Other malignancy	0	0	0	0	0	1/21 (5%)	0/1 (0)	0/1 (0)	0/1 (0)	0
Benign tumor	0	0	0	0	0	3/21 (14%)	0/2 (0)	0/2 (0)	0/3 (0)	0/1 (0)
Inconclusive report	0	0	0	0	0	5/21 (24%)	0/4 (0)	0/4 (0)	1/5 (20%)	0/3 (0)
Missing FNA report	1/11 (9%)	0/1 (0)	0	0/1 (0)	0	0	0	0	0	0

**Effusions (n = 29)**	**TB (n = 19)**					**NON-TB (n = 10)**				
	Cytology	ZN	Culture +	GeneXpert +	MPT64 +	Cytology	ZN	Culture +	GeneXpert +	MPT64 +
**Epitheloid cells + / − mixed inflammatory cells and + / − necrosis**	0	0	0	0	0	0	0	0	0	0
**Lymphocytes and/or (foamy) macrophages**	12/17 (71%)	0/11 (0)	4/11 (36%)	3/11 (27%)	8/10 (80%)	2/10 (20%)	0/2 (0)	0/2 (0)	0/2 (0)	0/1 (0)
**Mixed inflammatory cells with necrosis**	3/17 (18%)	0/3 (0)	1/1 (100%)	1/3 (33%)	3/3 (100%)	0	0	0	0	0
Mixed inflammatory cells without necrosis	2/17 (12%)	0/2 (0)	2/2 (100%)	2/2 (100%)	2/2 (100%)	2/10 (20%)	0/1 (0)	0/2 (0)	0/2 (0)	0/1 (0)
Malignancy	0	0	0	0	0	1/10 (10%)	0/1 (0)	0/1 (0)	0/1 (0)	0/1 (0)
Inconclusive result	0	0	0	0	0	5/10 (50%)	0/4 (0)	0/5 (0)	0/5 (0)	0/2 (0)
Missing cytology report	2/19 (11%)	0/2 (0)	1/2 (50%)	1/2 (50%)	1/1 (100%)	0	0	0	0	0

Findings compatible with TB in bold.

An independent reading of the immunostaining of 20 slides was performed by LS to assess the quality of the reading of the test. There was agreement on 11 positive MPT64 slides, 2 had a discordant result, and 7 slides were labelled by LS as inconclusive. Only 1/7 was also marked as inconclusive by the local pathologists.

## Discussion

This was a real-life study in the high TB and HIV prevalence setting where the MPT64 test was implemented in the routine EPTB diagnostics and used by the clinicians. The MPT64 test had a sensitivity of 92%, and performed far better than conventional tests such as Mtb culture and the WHO recommended GeneXpert in children with varied EPTB presentations. This is the second study where we have shown that the test is implementable in a basic pathology laboratory facility in a low-resource setting^7^. The results are consistent with results in the more controlled setting and shows that the MPT64 test can help in diagnosing pediatric EPTB^5^.

Our sample size is small with 30 EPTB patients in total. It is therefore difficult to make a good assessment of performance for different sites of disease. A recent study in Zanzibar found a sensitivity of the MPT64 test of 100% for pediatric TB adenitis^7^. In our study the risk for false negative MPT64 test results was low, which highlights its potential role as a confirmatory test for EPTB, adding sensitivity and timeliness to Mtb culture. As only 16 non-TB samples was assessed by the MPT64 test, the specificity (88%) could have been both under- or overestimated. Three non-TB samples had an uncertain MPT64 result, and were removed from the analysis of specificity. A conclusive result on these samples could have both increased or decreased the specificity of the MPT64 test.

The quality assessment performed by LS shows that many slides (7/20) were labelled as inconclusive by an outside pathologist from a Norwegian university hospital. 5/7 were labelled as uncertain due to poor quality of the slide. However, the pathologists in Mbeya Zonal Referral Hospital that had assessed over 250 MPT64 slides stained locally, did not report this ambiguity. This underlines that the result of the MPT64 test is dependent on the reader, and familiarity with local variations in staining patterns and artifacts.

Mtb culture performed poorer than the MPT64 test, but the assessment of its performance could have been affected by the number of contaminated samples. 10 Mtb culture samples were contaminated, of which 7 among TB patients. The overall sensitivity of Mtb culture on 22 samples was 45%. The transportation of samples to the Central Tuberculosis Reference Laboratory in Dar es Salaam could have affected the performance of Mtb culture both positively and negatively. However, being necessary for drug susceptibility testing, the long turn-around time and poor sensitivity of the test makes it less feasible as a stand-alone test. The poorer sensitivity of Mtb culture of 13% for EPTB samples is confirmed in our Zanzibar study^7^. Mtb culture remains an important diagnostic test despite its limitations and efforts should be made to improve the method through better laboratory facilities.

GeneXpert had a lower sensitivity of 31% as compared to Mtb culture (45%). Two samples (20%) were Mtb culture positive and GeneXpert negative. In previous studies GeneXpert has been shown to have a comparable, but slightly lower sensitivity than Mtb culture for various EPTB samples^1,2^. The advantage of GeneXpert compared to Mtb culture is the turn-around time, and in this study the reduced risk of inconclusive or contaminated results. A positive test result is very useful due to the high specificity, but the risk of false negatives is high, reducing its role as a stand-alone test for EPTB. The cost of GeneXpert when not subsidized is also a source for sustainability concerns^20^.

FNAC performed excellent in this study with a sensitivity of 90% and a specificity of 95%. These findings support the use of this simple test for diagnosis of EPTB. FNAC is simple, can be performed decentralized, slides can be prepared and sent to a central laboratory. However, due to the small sample size in our study (10 TB and 21 non-TB samples), the results should be interpreted with caution. FNAC has been shown to be unspecific in other settings, and diseases such as sarcoidosis, non-tuberculous mycobacteria and other granulomatous conditions can mimic TB^3^. The MPT64 antigen detection test can be used as a confirmatory test in cases where there is a suspicion for other granulomatous conditions. The pathologists did not report a suspicion of any non-TB granulomatous conditions in this study, which is similar to the results from Zanzibar. The MPT64 test had a correlation of 100% with positive FNAC results in our study. One FNAC sample did not show typical features of TB adenitis, but was MPT64 positive. The results from Zanzibar also supports a potential use of MPT64 as a confirmatory test, separating TB from non-TB related necrosis in lymphadenitis.

Cytological findings in cell smears of the effusions lacked both specificity and sensitivity to diagnose EPTB. The MPT64 test was positive in 14/16 TB cases that had effusions sampled, indicating the utility of the test in various types of samples. However, due to a small sample size, the results need to be validated in further studies.

The superior performance of MTP64 compared to Mtb culture and GeneXpert can be attributed to the paucibacillary nature of EPTB, and the potential role of antigen accumulation as a central part of EPTB pathogenesis. Antigen accumulation, rather than the bacillary load has been described as the central phenomenon in TB pathogenesis and tissue destruction^21,22^.

Accounting for the fact that Mtb culture is an imperfect gold standard, the MPT64 test was compared to a composite reference standard (CRS). We have included response to treatment to TB treatment as a part of the CRS to mimic real life practice. However, response to treatment is not conclusive as especially Rifampicin is fairly wide-spectred and would treat many conventional bacterial infections. The CRS accounted for this uncertainty by labeling patients with grades of certainty of their diagnosis; confirmed, probable, possible. The use of a CRS comes with a risk of misclassification bias. The MPT64 test was implemented and used by the clinicians, and some patients were started on TB treatment on the basis of their MPT64 result. If MPT64 had a tendency of being false positive in other infectious conditions, these patients could have been misclassified as TB cases. However, only two TB adenitis cases have been labelled as TB cases on the basis of response to treatment. One of these cases was MPT64 positive, and the other had a missing MPT64 test result.

In our study the prevalence of peritonitis (30%) and meningitis (20%) was higher than the usual distribution of pediatric EPTB sites. The severe presentations do not seem to be explained by a lack of BCG coverage, as BCG coverage was very good in our cohort. With concomitant malnutrition the severe presentations of TB peritonitis and meningitis is not surprising, but is not well documented in other studies^23^. Immunosuppression due to malnutrition, young age among the malnourished and recruitment as inpatients could have been contributing factors.

The prevalence of malnutrition was very high (29%). Malnutrition was more prevalent among the TB patients, as compared to the non-TB group, highlighting the need for a high level of clinical suspicion of not only pulmonary, but also extrapulmonary TB in this patient group. This is supported by a recent study in Ethiopia showing a higher incidence of malnutrition in adults with EPTB than in those with PTB^24^. From previous research it appears that malnutrition is a predictor of tuberculosis disease and is associated with worse outcomes^23,25^. In our study, 13/17 (76%) of the malnourished patients had EPTB, with peritonitis (4 patients) and meningitis (4 patients) being the most prevalent. Only 1/8 HIV infected patients had malnutrition, which reduces its potential impact on malnutrition in this study. Three of the malnourished patients had malignancies as their final diagnosis, which highlights that the malnourished patients in this study had serious concomitant diseases**.**

Both Mtb culture and GeneXpert performed poorer in the malnourished, with sensitivities of 30% and 25%, respectively. The MPT64 test had a sensitivity of 90%, but the results have to be interpreted with caution as only 10 samples were asessed. Also, surprisingly, the sensitivity of Mtb culture and GeneXpert did not improve in immunosuppressed patients (sensitivity of 38% and 28% respectively), where one would expect that the poor immune response of the host would lead to a greater bacterial load.

The mortality rate was equally high in the TB and non-TB group (23% vs 21%). However, these results could have been affected by the high number of patients lost to follow up in the non-TB group (13% vs 39%, TB versus non-TB group, respectively), masking a potential lower mortality rate in the non-TB group. When looking at mortality through the lens of malnutrition, one sees a trend towards increased mortality in this group with a mortality rate of 36%. A recent systematic review from Ethiopia found a double incidence of death in children < 5 y with severe acute malnutrition (SAM) and concomitant TB, and a high case fatality rate of SAM at 11.3%^26^. However, it is not specified if cases of EPTB are included in the review and their potential contribution to excess mortality. The contribution of TB to excess mortality in pediatric SAM is also reproduced in Zambia with a case fatality rate among children with SAM and TB of 56%. However, 94% of the TB cases in the Zambian study were pulmonary and the HIV prevalence was higher at 46.5% among SAM patients. The low number of EPTB cases in this study could point towards under detection^27^. Estimating the mortality rate of pediatric EPTB is challenging due to the lack of uniform case definitions and lack of standardized follow ups. Unfortunately, our small sample size reduces the strength of the analysis of contributing factors to mortality.

The trend of increased mortality was stronger in the group that included all causes of immunosuppression. In total, 10/61 patients died, of which 8 had immunosuppression. 6 patients that died had TB, 3 had malignancies. This highlights the need for a comprehensive evaluation of patients to ensure that various reasons of immunosuppression are considered.

Mbeya Zonal Referral Hospital is a tertiary referral hospital with laboratory services, radiology services and a histopathology lab. In this setting 51% of the presumptive EPTB cases were not classified as TB cases. In an even more resource constrained setting these patients could have been subject to a trial of TB treatment on suspicion of EPTB, leading to unnecessary treatment and diagnostic delay or even a wrong diagnosis. In our study the potential differential diagnosis ranged from benign conditions to life threatening conditions. 11/61 (18%) were diagnosed with malignancies and 10/61 (16%) with other infectious conditions. This highlights the need for diagnostic facilities and an awareness of differential diagnosis when examining patients with suspected pediatric EPTB. Under TB control programs only treatment for EPTB is free of cost, but all the diagnostic work-up incurs great financial burden on the patients. Diagnostic delay could have been a contributing factor to the high mortality. Early detection and timely referral of presumptive EPTB from local clinics to the tertiary hospital is probably needed to ensure a correct diagnosis and to reduce mortality.

In conclusion, the MPT64 test was implementable in the routine TB diagnostic setting in a tertiary hospital in a low-resource, high TB and HIV setting, and the test performance was better than routine diagnostic tests, including GeneXpert. However, this test cannot be implemented until a basic laboratory structure is available and health systems are strengthened. The method of immunostaining used in the MPT64 test is also useful for strengthening diagnosis of other diseases, especially cancers.

## Supplementary Information

Supplementary Information 1.

Supplementary Information 2.

## Data Availability

All data generated or analyzed during this study are included in this published article (and its Supplementary Information files S1 Dataset and S2. Study questionnaire.).
