# Supplementary material for: MPT64 antigen detection test improves diagnosis of pediatric extrapulmonary tuberculosis in Mbeya, Tanzania
Source: Sci Rep. 2021 Sep 2;11:17540. doi: 10.1038/s41598-021-97010-2 (PMC8413277; doi:10.1038/s41598-021-97010-2)
Supplement: Supplementary file 2 — Supplementary Information 2. [file 41598_2021_97010_MOESM2_ESM.pdf]

NATIONAL INSTITUTE FOR MEDICAL RESEARCH
MUHIMBILI MEDICAL RESEARCH CENTRE
P.O.Box 3436, Dar es Salaam, Tanzania.

IRRSSIA STUDY
Score chart for diagnosis of TB in children.

SCORE IF SIGN OR SYMPTOM PRESENT						
	**0**	**1**	**2**	**3**	**4**	**Score**
**General features**						
Duration of illness	Less than 2 weeks	2-4 weeks		More than 4 weeks		
Failure to thrive or weight loss	Weight gain	No weight gain or weight faltering		Weight loss		
TB contact	None	Reported (but no documentation) smear-negative or EPTB		Smear positive (with documentation)		
TST	Negative, not done			Positive		
Malnutrition not improved after four weeks of therapy				Present		
Unexplained fever not responding to appropriate therapy			Positive			
**Local features**						
Chest x-ray				TB-suggestive features like infiltration, cavity, or hillar lymph nodes		
Painless, enlarged lymph nodes		Any non-cervical lymph nodes		Positive cervical lymph nodes		
Swelling of bones or joints				Positive		
Unexplained ascites or abdominal mass				Positive		
Central nervous system findings: meningitis, lethargy, irritability and other behaviour changes				Positive		
Angle deformity of the spine					Positive	
**TOTAL SCORE** A score of **7 or more** indicates a high likelihood of TB. Refer the child for TB treatment.						

Adopted from the NTLP manual for TB diagnosis, 6th Edition of 2013

NATIONAL INSTITUTE FOR MEDICAL RESEARCH
MUHIMBILI MEDICAL RESEARCH CENTRE
P.O.Box 3436, Dar es Salaam, Tanzania.

CRF 03

IRRSSIA STUDY FORM II

Majibu kutokana na uchunguzi wa sampuli za mgonjwa. Jaza fomu tofauti kwa kila sampuli kutoka kwa mgonjwa

LABORATORY INVESTIGATIONS

Namba ya Utambulisho ya Mradi ____________________ Tarehe:____/____/_______.

Namba ya Mgonjwa ya Hospitali _____________________

HP No.: _______________ CP/HC No.:________________

Age:_____ Sex:____

Number of samples collected:_________

Sample material:

☐ Pleura fluid ☐ Lymph node biopsies ☐ Cerebrospinal fluid
☐ Ascites ☐ Fine needle aspirates ☐ Other: ______________________
☐ Tissue

Lymph Node Site: ☐ Cervical ☐ Inguinal ☐ Other: __________________
☐ Axillary ☐ Mediastinal

AFB microscopy: ☐ Positive ☐ Negative

Cytology/histology:

____

____

____

MTP64: ☐ Positive ☐ Weak Positive ☐ Negative ☐ Uncertain

TAARIFA MUHIMU:weka alama kama sampuli imepatikana.

☐ Sampuli kwaajili ya culture imepatikana na kupelekwa maabara CTRL ☐ Hapana
☐ Sampuli kwaajili ya GeneXpert imepatikana na kupelekwa CTRL ☐ Hapana
☐ Sampuli ya damu imepatikana na kutengeneza DBS and DPS ☐ Hapana

NATIONAL INSTITUTE FOR MEDICAL RESEARCH
MUHIMBILI MEDICAL RESEARCH CENTRE
P.O.Box 3436, Dar es Salaam, Tanzania.

IRRSSIA STUDY FORM III

(fomu ya sampuli kwaajili ya MTB Culture and GeneXpert at CTRL)

Namba ya Utambulisho ya Mradi ______________________ Tarehe:____/____/_______.

Namba ya Mgonjwa ya Hospitali ______________________

Sample received from: ______________________________

Sample material:

- ☐ Pleura fluid
- ☐ Lymph node biopsies
- ☐ Ascites
- ☐ Fine needle aspirates

Date sample collected: _________________(dd/mm/yy)

Volume of sample received: ________ml

Date sample tested: __________________________________

MTB Culture: ☐ Positive ☐ Negative ☐ Contamination

GeneXpert: ☐ Positive ☐ Negative ☐ Invalid

Rif resistance: ☐ Yes ☐ No

QUESTIONNAIRE – CHILDREN BELOW THE AGE OF 18 (CRF 01)

Unique Study Number: ____________________ **Date:** ____________

Consultant: ____________________

Hospital:

☐**Muhimbili National hospital**

☐**Mbeya Referral Hospital**

☐**Other**

Department: ☐ OPD ☐ IPD

Is the patient on TB drugs? Yes. No

For outpatients, from which hospital was the patient referred? ____________________

Hospital patient number of the referring hospital: ____________________

PATIENT IDENTIFICATION

Name of patient:____________________ **Study Number**: ____________

Age:________(years) Mobile#: ____________________

Close relative contact (1):____________________Close relative contact (2): ____________________

Gender: ☐ Male ☐ Female

1. **Respondent:** ☐ Patient ☐ Parent ☐ Spouse ☐ Child ☐ Other, relative/friend

Address: Region__________________Village________________Street______________________

2. **Living with:**

☐ one parent (mother/father) ☐ both parents ☐ Relatives ☐ Orphanage
☐ Others, please specify____________________

3. **Education/day-care:**

☐ home-care ☐ nursery ☐ primary school
☐ Form I-IV ☐ Form IV-VI ☐Others (Please mention) ______

4. **Religion**:

☐Muslim ☐ Christian ☐ Other, please mention___________

PERSONAL AND PAST MEDICAL HISTORY

5. **Tobacco use (adolescents):** □ Yes □ No ____weeks/months /years
6. **Smoking (adolescents):** □ Yes □ No ____weeks/months /years
7. **Alcohol (adolescents):** □ Yes □ No ____weeks/months /years

8. **Associated Diseases**

Asthma: □ Yes □ No
Chronic diarrhoea □ Yes □ No
Renal Disease: □ Yes □ No
Liver Diseases: □ Yes □ No
Diabetes Mellitus: □ Yes □ No
Cardiac disease: □ Yes □ No
Other: □ Yes □ No
Describe other: ______________________________
____

9. **Medication**: ***Please write the names of the medication***

____

PAST HISTORY OF TUBERCULOSIS

10. **Has the child been in contact with a person with known tuberculosis?**

□ Yes □ No

If yes, who was/were the contact person(s)?

____

If yes, when was the child in contact with this (these) person(s)?

____

11. **Has the child previously been diagnosed with pulmonary tuberculosis?**

□ Yes □ No

12. **Has the child previously been diagnosed with extrapulmonary tuberculosis?**

□ Yes □ No

13. **Has the child previously been treated for tuberculosis?**

□ Yes □ No

14. **If the child has been treated, what was the treatment outcome?**

□ Cured
□ Treatment Completed
□ Treatment Interrupted

15. **If treated, when was the last time the child completed any TB treatment?_________________**

HEALTH-SEEKING BEHAVIOUR

Health seeking behavior for TB patients
Please remind the patient and respondent that this survey is confidential.

16. Please ask if the patient has experienced any of the following symptoms

General Symptoms

Fever: ☐ Yes ☐ No ____weeks/months

What kind of fever does the child have?

☐ High-grade ☐ Low-grade

When does the child have fever?

☐ Morning ☐ Day-time ☐ Evening ☐ Night ☐ all day

Failure to gain weight ☐ Yes ☐ No ____weeks/months

Loss of weight: ☐ Yes ☐ No ____weeks/months

Loss of appetite: ☐ Yes ☐ No ____weeks/months

Night Sweat: ☐ Yes ☐ No ____weeks/months

Fatigue: ☐ Yes ☐ No ____weeks/months

Body weakness: ☐ Yes ☐ No ____weeks/months

Frequent cold: ☐ Yes ☐ No ____weeks/months

Neck mass: ☐ Yes ☐ No ____weeks/months

Other: ☐ Yes ☐ No ____weeks/months

Respiratory Symptoms

Cough: ☐ Yes ☐ No ___weeks/months

Sputum: ☐ Yes ☐ No ___weeks/months

Cough with Sputum: ☐ Yes ☐ No ___weeks/months

Cough with blood: ☐ Yes ☐ No ___weeks/months

Chest pain: ☐ Yes ☐ No ___weeks/months

Difficult in breathing: ☐ Yes ☐ No ___weeks/months

Abdominal Symptoms

Swelling of/in stomach: ☐ Yes ☐ No ___weeks/months

Fullness of the stomach: ☐ Yes ☐ No ___weeks/months

Vomiting: ☐ Yes ☐ No ___weeks/months

Chronic diarrhea: ☐ Yes ☐ No ___weeks/months

Other: ☐ Yes ☐ No ___weeks/months

Describe other: ____

____

\

Neurological Symptoms

Headache: ☐ Yes ☐ No ___weeks/months

Irritability ☐ Yes ☐ No ___weeks/months

Photophobia: ☐ Yes ☐ No ___weeks/months

Vomiting: ☐ Yes ☐ No ___weeks/months

Dizziness: ☐ Yes ☐ No ___weeks/months

Vertigo: ☐ Yes ☐ No ___weeks/months

Weakness/Numbness of extremity:

☐ Yes ☐ No ___weeks/months

Visual disturbance: ☐ Yes ☐ No ___weeks/months

Other: ☐ Yes ☐ No ___weeks/months

Describe other: ______________________________

____

____

17. What were the major symptoms that first made you seek care for your child?

☐ Prolong Cough ☐ Coughing blood ☐ Breathlessness
☐ Chest pain ☐ Fever ☐ Weight loss
☐ Fatigue\Weakness ☐ Loss of appetite ☐ Night sweats
☐ Bone pain ☐ Lymph node swelling ☐ Diarrhoea
☐ Abdominal pain ☐ Others (specify) _________________

18. When did you or the child first notice the symptoms?

____

19. Did you give the child any self-medication before you sought care?

☐ Yes ☐ No

20. When did you first seek medical advice for the child after noticing the symptoms?

Mention the exact date: ___/___/______

(If you don't remember the exact date but it was on the beginning of the month fill 05, if it was in the middle of the month fill 15 and if it was on the end of the month fill the 25).

21. How many different places did you go to seek help for the child's current symptoms?
________________places

22. How many times have you taken the child to a health facility with the same symptoms before?

☐ First visit ☐ Second visit ☐ Third visit
☐ > 3 visits ☐ don't remember

23. Which place did you first seek care for the child`s symptoms?

☐ Regional Hospital ☐ District hospital ☐ Health center
☐ Dispensary ☐ Private Hospital ☐ Traditional healer
☐ Pharmacy ☐ other, please specify____________________________________

24. Did the child get any medicine from there?

☐ Yes ☐ No

25. If yes, what kind of medicine?

☐ Antibiotics ☐ Anti-TB ☐ Herbs ☐ others, which_____________

26. Were the child`s symptoms relieved after taking medicines?

☐ Yes ☐ No

27. What kind of diagnosis did the child receive for the current illness?

____

28. Were any tests done at the first medical service?

☐ Yes ☐ No

29. What type of tests?

☐ Blood test ☐ Urine test ☐ Sputum ☐ X-ray

□Others, please specify________________________________

30. Did you take the results back to the doctor?

□ Yes □ No

31. Could you estimate the total cost for the previous visits/investigations related to the child`s current illness?

Admission __________________________TZS

Consultations __________________________ TZS

Medication __________________________TZS

Laboratory tests/X-ray/CT_________________________TZS

Transportation __________________________TZS

□ All costs covered by Medial Insurance

32. Who referred the child here to this health facility?

□ myself □ Traditional healers □ Religious leaders

□ Pharmacy/drug shop □ Village health worker □ Government dispensary

□ Government health center □ Government hospital □ Private dispensary/hospital

□ Charitable/NGO □ Member of the family □ Other____________________

33. Has your child received routine vaccination, by following the national children`s vaccination program?

□ Yes □ No

34. Has your child received BCG vaccination?

□ Yes □ No

35. Has the child ever been tested for HIV?

□ Yes □ No

36. What was the result of the HIV-test?

□ HIV positive □ HIV negative □ don't know □ don't agree to disclose HIV status

37. Before today, had you heard of the illness tuberculosis?

□ Yes □ No

38. Do you know any symptoms of tuberculosis?

□ Chronic cough □ Spitting blood □ Shortness of breath

□ Chest pain □ Fever □Weight loss

□ Tiredness □ Loss of appetite

□ Others Please specify______________________________ ***(Do not probe but ask for more symptoms)***

39. Do you know which parts of the body that can be affected by tuberculosis?

____

40. Can tuberculosis spread from person to person?

□ Yes □ No □ Uncertain

41. In your household do you drink unboiled milk?

□ Yes □ No

42. In your household do you eat raw meat?

□ Yes □ No

43. Did you know that consumption of raw animal products, like uncooked dairy products can lead to gastrointestinal tuberculosis as a result of transfer of the disease from animals to humans?

 Yes No

44. Can tuberculosis be cured with medicines?

 Yes No Uncertain

45. Do you know how long it takes to treat tuberculosis?

 Yes No

If yes, do you know the approximate duration of treatment? ______________________

46. Do people in your community associate tuberculosis with HIV?

 Yes No Uncertain

If yes, why do they associate it with HIV? __________________________________

47. Is there anything that would make it easier for people with tuberculosis to get treatment, not just in this clinic, but in other health facilities?

 Yes No Uncertain

If yes, what could be done? ____

48. The moment you realized that the child may have contracted tuberculosis, did you have any problems deciding to seek care? If so, what types of problems?

____

____

____

49. What fears do others have about TB that prevents them from seeking medical advice?

____

50. If you consulted a traditional healer before seeking care at a modern health facility, what were the reasons which led you to first use the traditional healer?

____

PATIENT AND HOUSEHOLD COSTS, estimate of the patient income level

51. How long does it take you to go to the nearest health facility?

 Less than 30 minutes between 30 minutes and one hour More than one hour

52. How far is this hospital to your home (in Kilometers) _________________

53. How long (on average) does it take you to this health facility, waiting for the child`s consultation and finally returning to your home\workplace? ________________ Hours

54. How did you get to this health facility?

 Walked Bicycle Motorcycle Private car Dala Dala

55. If you have to take a Dala Dala, how much (on average) does it cost you to come to the clinic? _______________TZS.

56. Do you usually have to make some special arrangements at home before coming to the clinic?

☐ Yes ☐ No ☐ Uncertain

If yes, what arrangements? _________________________

57. What is the main income of your household?

☐ Crop production ☐ Livestock ☐ Fishing
☐ Hunting/ bee-keeping ☐ Poultry ☐ Farm wage
☐ Other agricultural activity ☐ Wages (government) ☐ Wages (private)
☐ Monetary savings (interest) ☐ Pensions
☐ Property (rentals) ☐Self-employed payments (merchant)
☐Other Specify___________________________

58. In the past 12 months, in what types of activities were you and any members of your household engaged? (Only income-generating activities)?

____
____
____

59. How much did (NAME) earn (money) for the activities stated on average in the past 12 months? This should include not only salary or cash income: but also the value of goods produced or traded for other goods and services.

____

60. Have any member of your household stopped working or reduced their work capacity because of the child`s illness?

☐ Yes ☐ No

If yes, for how long? ______________days

If yes, how much reduced working capacity? _________________________________

61. Have you/or any member of your household lost any wages or income because of the child`s illness?

☐ Yes ☐ No ☐ Uncertain

If yes, how much___________________

62. Do you own a house?

☐ Yes ☐ Renting a house ☐ living with relatives /friends ☐ Homeless

63. How many people live in your household: ________________ (number of people)

How many: Men: _______Women:_______Elderly:_______Children (between 0-10):_______
Children (between 11-18): _______

64. How many siblings does the child have?__________sibling(s)

65. What is the main source of drinking water for members of your household?

☐ Piped water 1=Piped into dwelling 2= Piped into yard/plot 3=Public tap 4=Neighbors' tap
☐ Water from open well
☐ Water from covered well or borehole
☐ Running water 1=spring; 2=river/stream; 3=pond/Lake; 4=Dam
☐ Rain water

☐ Tanker truck
☐ Water vendor
☐ Bottled water
☐ Others Specify ________________________

66. What kind of toilet facilities does your household have?

☐ Flush toilet ☐ Pit toilet/latrine 1=traditional pit latrine 2=ventilated pit latrine (VIP)
☐ No facility/bush/field ☐ other, please specify ______________

67. Do you share these facilities with other households?

☐ Yes ☐ No

68. Does your household have?

☐ Electricity ☐ Paraffin lamp ☐ Radio
☐ Television ☐ Telephone/mobile ☐ Iron (either charcoal or electricity)
☐ Refrigerator

69. What is the main source of energy for lighting in your household?

☐ Main electricity ☐ Solar ☐ Gas
☐ Paraffin-hurricane lamp ☐ Paraffin-Wick lamp ☐ Firewood
☐ Candles ☐ other, please specify _________________________

70. What is the main material for the walls of your house or house you are living?

☐ Grass ☐ Poles and mud ☐ Cement bricks
☐ Backed bricks ☐ Timber ☐Stones
☐ Others Specify_________________________

71. What is the roofing material of your house or house you are living?

☐ Grass/leaves/mud ☐ Iron sheets ☐ Tiles ☐ Concrete ☐ Asbestos
☐ Others Specify_______________

72. Does any member of your household own

☐ A bicycle ☐ A motorcycle or motor scooter ☐ A car ☐ A bank account

73. How many acres of land for farming/grazing are owned by the household?

☐ Arable land_____________acres ☐ Land for grazing__________acres

74. How many meals does your household usually have per day?

Meals_________________

NATIONAL INSTITUTE FOR MEDICAL RESEARCH
MUHIMBILI MEDICAL RESEARCH CENTRE
P.O.Box 3436, Dar es Salaam, Tanzania.

IRRSSIA STUDY FORM I

Sehemu hii inajazwa na anaejaza dodoso la mgonjwa. Taarifa kutoka kwenye file la mgonjwa

Namba ya Utambulisho ya Mradi ______________________ Tarehe:____/____/_______.

Namba ya Mgonjwa ya Hospitali _______________________

Age:_____ Sex:_____

Hb _____ ESR ____ WBC count: ____

HIV: ☐ Positive ☐ Negative ☐ Unknown

Biochemical tests:

☐ Protein: __________ ☐ Glucose: __________ ☐ Cell count: ____________

PHYSICAL SIGNS:

Weight: ______kg. Temperature: ______ ^{0}C.

Pulse rate: ______bpm BP:__________mmHg MUAC _________cm

Pallor: ☐ Yes ☐ No
Finger clubbing: ☐ Yes ☐ No
BCG scar: ☐ Yes ☐ No
Other: ☐ Yes ☐ No

LYMPH NODES:

Lymph node enlargement: ☐ Yes ☐ No
Matted: ☐ Yes ☐ No
Painful: ☐ Yes ☐ No
Discharge/Sinus: ☐ Yes ☐ No

DRAW ENLARGED LYMPH NODES OR OTHER FINDINGS:

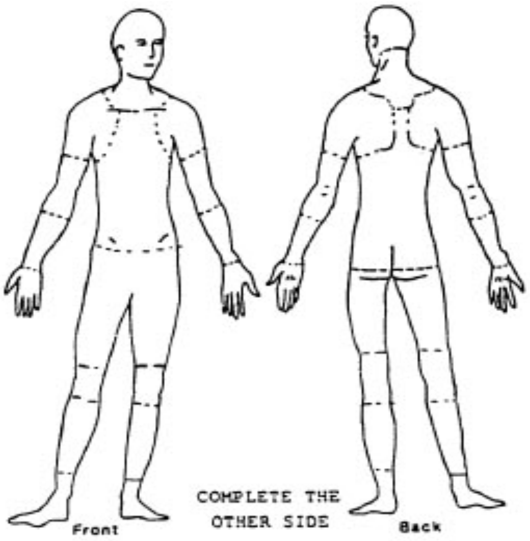

CHEST FINDING

☐ Yes: _____weeks/months ☐ No ☐ Not Done

If **YES** describe findings:

____

____

____

CARDIOVASCULAR SYSTEM

Murmur: ☐ Yes ☐ No ☐ Not Done

Other:

Describe Other:

____

____

____

ABDOMEN

Mass:	☐ Yes	☐ No	☐ Not Done
Ascites:	☐ Yes	☐ No	☐ Not Done
Hepatomegaly:	☐ Yes	☐ No	☐ Not Done
Splenomegaly:	☐ Yes	☐ No	☐ Not Done
Other:	☐ Yes	☐ No	☐ Not Done

Describe Others:

____

____

____

NEUROLOGICAL EXAMINATIONS

Level of consciousness:	☐ Alert	☐ Altered	☐ Stupor	☐ Coma
Neck Rigidity:	☐ Yes	☐ No		
Photophobia:	☐ Yes	☐ No		
Tone Upper limbs:	☐ Normal	☐ Abnormal		
Tone Lower limbs:	☐ Normal	☐ Abnormal		
Power Upper limbs:	☐ Normal	☐ Abnormal		
Power Lower limbs:	☐ Normal	☐ Abnormal		
Coordination:	☐ Normal	☐ Abnormal		
Exaggerated Reflexes:	☐ Yes	☐ No		
Other:	☐ Yes	☐ No		

Describe Others:

____

____

____

Other findings (Skin, Unilateral swelling/joint tenderness, backache, stiffness, lump, deformity, limp)

____
____
____
____
____

SPUTUM EXAMINATION: (kama mgonjwa amechukuliwa makohozi)

AFB microscopy:

	Date:(dd/mm/yy).	Appearance *	Neg.	+	++	+++
☐ **Sample 1**						
☐ **Sample 2**						
☐ **Sample 3**						

*visual appearance (blood stained, muco-purulent, saliva)

MTB Sputum Culture: ☐ Positive ☐ Negative

Date of positive culture:(dd/mm/yy): ________________________

GeneXpert: (sputum) ☐ Positive ☐ Negative

Rif resistance: ☐ Yes ☐ No

BACTERIOLOGY:

Gram stain: ____________________ **Bact. Culture:** ____________________

Other tests: ____
____

OTHER INVESTIGATIONS

Chest X-Ray:

____
____
____

Sonography/CT scan:

____
____
____

Other:

____
____
____

NATIONAL INSTITUTE FOR MEDICAL RESEARCH
MUHIMBILI MEDICAL RESEARCH CENTRE
P.O.Box 3436, Dar es Salaam, Tanzania.

CRF 05

IRRSSIA STUDY FORM IV

(Information obtained from DTLC)

Namba ya Utambulisho ya Mradi ______________________ Tarehe:____/____/_______.

Namba ya Mgonjwa ya Hospitali _______________________

Extra pulmonary: ☐ Yes ☐ No
Extra pulmonary with pulmonary: ☐ Yes ☐ No
Pulmonary: ☐ Yes ☐ No

Date of diagnosis: (dd/mm/yy): ______________________
Date of result communicated to the patient: (dd/mm/yy): ______________________
Date of started treatment: (dd/mm/yy): ______________________

TB TREATMENT OUTCOME:

- ☐ Cured
- ☐ Treatment completed
- ☐ Died
- ☐ Treatment Interrupted
- ☐ Transfer Out

What is the patient final diagnosis: ____
____
